# Impact of a Multidisciplinary Head and Neck Tumor Board on Treatment and Survival in Laryngeal Carcinoma

**DOI:** 10.3390/curroncol30120733

**Published:** 2023-11-23

**Authors:** Katharina El-Shabrawi, Valentin Burkhardt, Christoph Becker

**Affiliations:** Department of Otorhinolaryngology, Head and Neck Surgery, Faculty of Medicine, Medical Centre, University of Freiburg, 79106 Freiburg, Germany

**Keywords:** multidisciplinary tumor board, laryngeal carcinoma, survival outcome

## Abstract

Background: Pretherapeutic discussion in the head and neck tumor board (HNT) has been mandatory at the University Medical Center Freiburg since 01/2015, and it is intended to contribute to a survival benefit through interdisciplinary decision making. Prior to 2015, an optional HNT existed in which mainly advanced tumor stages were discussed. The aim of this study was to determine the effect of a pretherapeutic HNT on treatment and survival in laryngeal cancer. Methods: A retrospective data analysis of 412 laryngeal carcinoma patients treated at the Head and Neck Cancer Center of the University Medical Center Freiburg between 01/2010 and 12/2020 was conducted. Differences regarding TNM status, UICC classification, tumor localization, gender and age at initial diagnosis, recurrence, secondary tumors, therapy, 5-year survival, and 5-year recurrence-free survival (5YSR/5Y-RFS) were assessed for therapy initiation with or without a pretherapeutic HNT. Results: In total, 314 patients underwent a pretherapeutic HNT, and 98 received therapy initiation without an HNT. The HNT group showed significantly more advanced T stages and UICC classifications (*p* < 0.001; *p* = 0.003) and more frequent primary chemo/radiotherapy (*p* < 0.001). There was no significant difference regarding 5YSR (43 vs. 47 months, *p* = 0.96) or 5Y-RFS (48 vs. 52 months, *p* = 0.16). The time between initial diagnosis and therapy initiation was significantly longer when an HNT was performed (38 vs. 20 days, *p* = 0.008). Conclusions: The HNT group showed significantly more advanced tumor stages, suggesting that even before it became mandatory, it was frequently used for interdisciplinary case discussion in more complex cases. Due to the small number of T3/4 patients in the non-HNT group, a survival advantage of an HNT cannot be validly demonstrated in our study. However, the HNT led to broader patient counselling regarding their therapy options. At the same time, a significant delay in therapy initiation could be seen, suggesting that workflows between diagnosis, HNT presentation, and therapy initiation should be optimized.

## 1. Introduction

Therapeutic management of head and neck carcinomas can be highly complex, especially in locally advanced tumor stages, and it often requires interdisciplinary, coordinated therapy procedures. In the case of laryngeal carcinoma, it is estimated that there are 184,600 new cases and 99,800 deaths per year worldwide [1]. Therapeutically, either primary surgical resection or primary radiotherapy is recommended in UICC stages I–II. In advanced tumor stages III–IV, the German S3 guideline suggests the combination of surgical therapy and adjuvant (chemo)radiotherapy or primary chemoradiotherapy [2]. In order to improve the interdisciplinary collaboration of the different specialties involved in therapy, the implementation of head and neck tumor boards (HNTs) has been established. Performing a pretherapeutic HNT should not only improve multimodal therapy management but also achieve cost effectiveness in cancer treatment [3]. Advanced tumor stages in particular are expected to benefit from an interdisciplinary case discussion [4].

The implementation of a pretherapeutic head and neck tumor board (HNT) is one of the key criteria for clinics to be certified as a head and neck tumor center in Germany. Certification as a head and neck tumor center requires that 95% or more of primary tumor cases are presented in a pretherapeutic tumor board with otorhinolaryngologists, oral maxillofacial surgeons, radiation oncologists, pathologists, radiologists, and hematology oncologists present [5]. The data suggest that certified centers optimize oncological therapy and improve survival through the presence of professional multidisciplinary teams [6]. The Head and Neck Cancer Center Freiburg, which is part of the Comprehensive Cancer Center Freiburg, achieved certification in 01/2015. Prior to 2015, an optional HNT existed, where mainly advanced tumor stages were discussed. The aim of this study was to determine whether the case presentation in a multidisciplinary pretherapeutic HNT changed survival rates and recurrence-free intervals in laryngeal carcinoma. Additionally, differences in therapy regimens and other epidemiologic factors were assessed.

## 2. Materials and Methods

A retrospective data analysis of 412 laryngeal carcinoma patients treated at the Head and Neck Cancer Center of the University Medical Center Freiburg with or without pretherapeutic HNT between 01/2010 and 12/2020 was performed. Data concerning age at initial diagnosis, gender, tumor localization (referring to the ICD code), TNM and UICC classification referring to the 7th edition, dates of the pretherapeutic tumor board presentation and therapy initiation, therapy regimen (including adjuvant therapy), date and type of tumor recurrence, secondary tumors, and date of last follow-up or death were gathered. Exclusion criteria were patients with externally histologically confirmed initial diagnosis or externally performed primary therapy. Furthermore, we excluded all patients who presented with a history of previous malignancy in the head and neck area other than laryngeal carcinoma. Differences between patients with and without pretherapeutic HNT presentation were assessed, and, in the following, they are referred to as the “HNT group” and the “non-HNT group.” The primary endpoint of this study was the 5-year survival rate.

Statistical analysis was performed using the statistical program SPSS (version 29.0). Metric variables were depicted by the arithmetic mean and associated standard deviation. Categorical variables were represented by absolute and relative frequencies. Group comparisons for metric variables with independent samples were performed using Student’s *t*-test. Survival times were defined from the time of therapy initiation, and they were calculated and illustrated using the Kaplan–Meier method. Survival was calculated as the 5-year survival rate (5-YSR), and recurrence-free survival was calculated as the 5-year recurrence-free survival (5Y-RFS). Differences between survival times were assessed using the log-rank test. Some parameters were further analyzed with respect to their influence on survival using forward Cox regression.

## 3. Results

### 3.1. Analysis of Epidemiological Data

From the 412 retrospectively analyzed patients with laryngeal carcinomas between January 2010 and December 2020, 314 were presented and discussed in a pretherapeutic HNT, and 98 received therapy initiation without prior HNT. Age and gender did not differ significantly between the groups (*p* = 0.21, *p* = 0.15). The majority of tumors in both groups were located in the glottis region (55% and 65%), followed by supraglottis in the HNT group (24% and 13%) and overlapping sites of the larynx in the non-HNT group (18% and 15%). When considering TNM status, the significantly more advanced T status in the HNT group is notable (*p* < 0.001) as well as the significantly higher percentage of UICC stages III-IV (48% vs. 28%, *p* = 0.003). Regarding N-, M-, and R-status as well as recurrence and secondary tumors, there was no statistically significant difference between the groups. All results are summarized in Table 1.

### 3.2. Treatment Regimen

**Figure 1:**
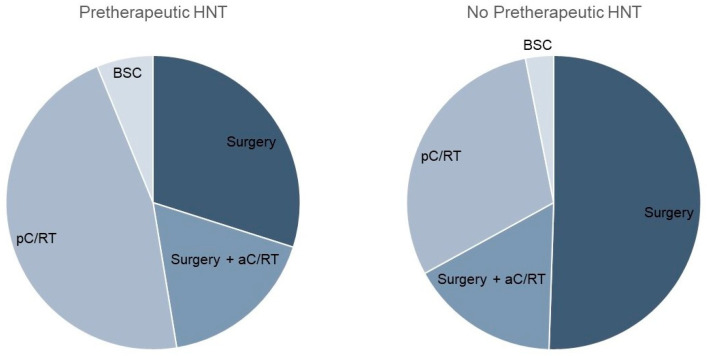
Comparison of therapy regimen with and without pretherapeutic tumor board presentation.

Considering the therapy regimen, we found a nearly equal distribution between surgery and primary C/RT in the pretherapeutic HNT group (Table 2; Figure 1). In contrast, the non-HNT group showed significantly more frequent primary surgical therapy (46% vs. 65%, *p* < 0.001). Adjuvant therapy was applied equally often in both groups (17% and 16%). The time between diagnosis and therapy initiation was significantly longer when a pretherapeutic HNT was held, amounting to 37.8 vs. 20.1 days, respectively (*p* = 0.008). As the preparation for primary C/RT is known to take longer than the preparation for surgical therapy, we analyzed these forms of therapy separately in a next step. Tumor board presentation resulted in a significant prolongation of time to therapy, with 20.6 vs. 11.8 days (*p* < 0.001) for surgically treated patients. For pC/RT, the time to treatment difference of 54.7 vs. 40.7 days was not significantly different (*p* = 0.4). The mean time between diagnosis and tumor board presentation was 12.3 days. In the HNT group, there were 18 cases that were not adherent to tumor board treatment recommendations. This was due to the patient’s request (*n* = 11), an intra-clinic decision (*n* = 4), or for unknown reasons (*n* = 3).

Because the HNT group showed significantly more advanced T stages, which, consequently, led to the more frequent use of pC/RT, differences in treatment regimen for T1/2 stages were calculated in a further step. Here, the significantly more frequent use of pC/RT after performing an HNT was demonstrated (31% vs. 19%, *p* = 0.027; Table 3).

### 3.3. Survival Analysis

**Figure 2:**
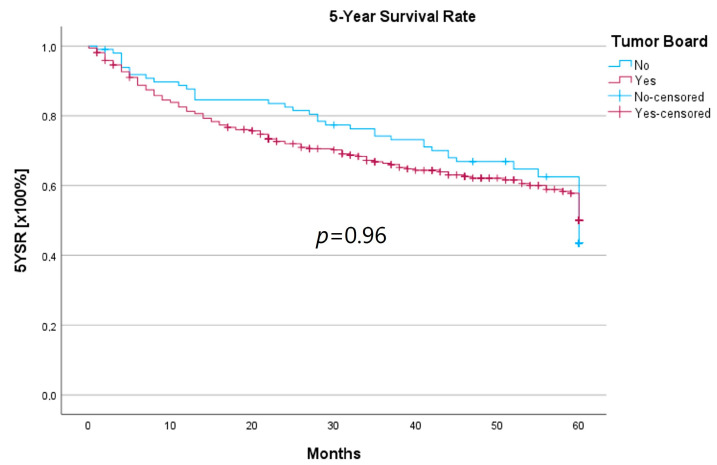
Comparison of the 5YSR for therapy initiation with or without prior HNT.

**Figure 3:**
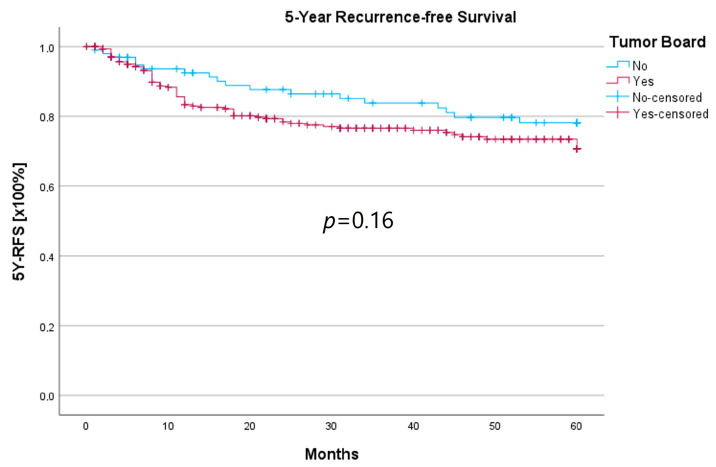
Comparison of 5Y-RFS for therapy initiation with or without prior HNT.

Survival was calculated as the 5-year survival rate (5YSR), and recurrence-free time was calculated as the 5-year recurrence-free survival (5Y-RFS), respectively. Upon comparing both groups, there was no statistically significant difference regarding 5YSR or 5Y-RFS between the HNT group and the non-HNT group (Table 4; Figure 2 and Figure 3).

**Figure 4:**
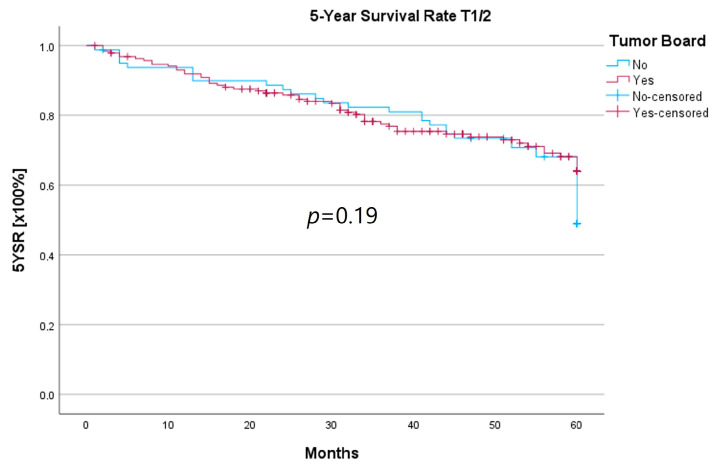
Comparison of 5YSR for T1/2 laryngeal carcinoma with or without pretherapeutic HNT.

**Figure 5:**
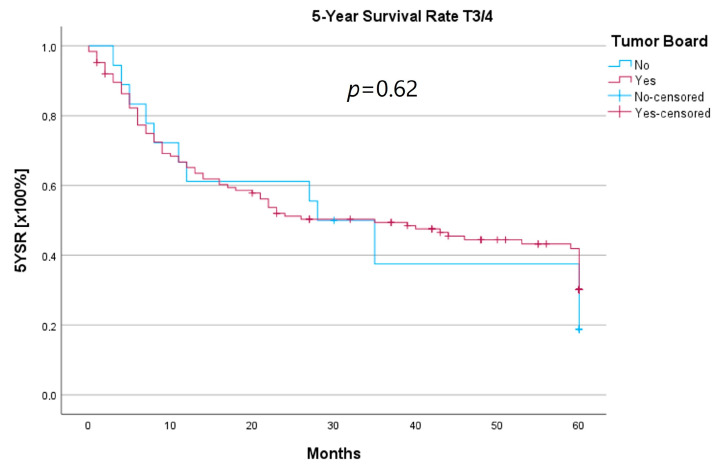
Comparison of 5YSR for T3/4 laryngeal carcinoma with or without pretherapeutic HNT.

**Figure 6:**
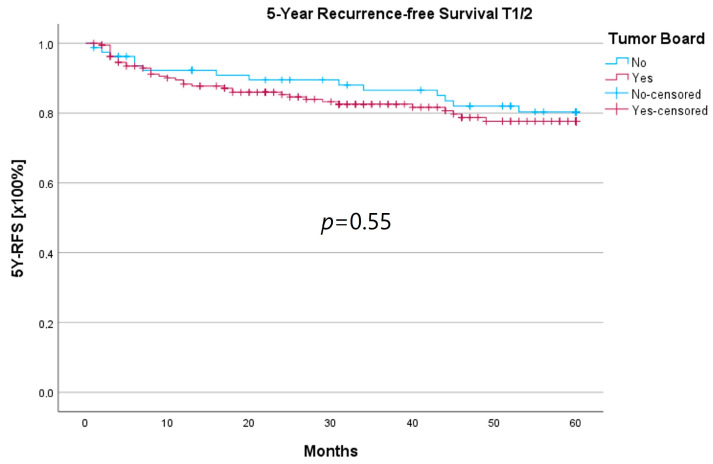
Comparison of 5Y-RFS for T1/2 laryngeal carcinoma with or without pretherapeutic HNT.

**Figure 7:**
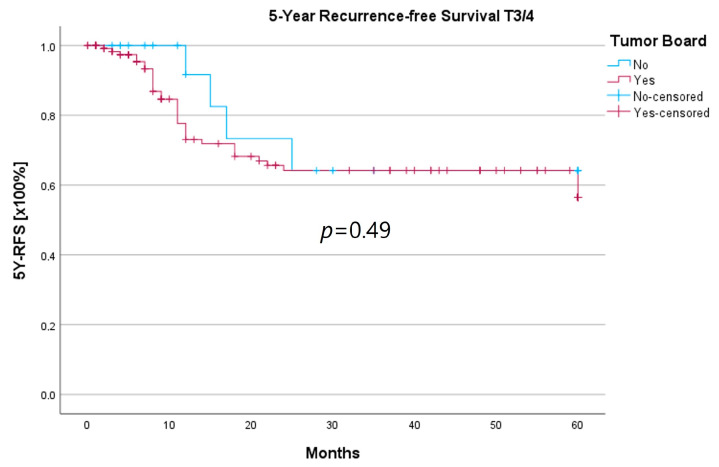
Comparison of 5Y-RFS for T3/4 laryngeal carcinoma with or without pretherapeutic HNT.

Because the HNT group also showed significantly advanced T stages, 5YSR and 5Y-RFS were calculated for T1/2 and T3/4 stages separately (Table 5). The mean 5YSR for T1/2 laryngeal carcinoma amounted to 49.7 months in the HNT group and 50.2 months in the non-HNT group (Figure 4). For T3/4 laryngeal carcinoma, the HNT group showed a slightly better 5YSR, with 33.7 months versus 32.7 months, respectively (Figure 5). Considering 5Y-RFS, the non-HNT group showed longer recurrence-free intervals for T1/2 carcinomas (50.8 vs. 52.9 months; Figure 6) as well as for T3/4 carcinomas (42.6 vs. 44.7 months; Figure 7). However, the differences between the groups cannot be regarded as statistically significant.

In order to identify factors that had a significant impact on survival, a forward Cox regression analysis was performed. Here, age at initial diagnosis was identified to exert the greatest influence on survival outcomes, followed by UICC classification and the number of secondary malignancies (Table 6). In the observed data, there was a 5.1% increase in the risk of death with a one-year increase in age. In this model, no statistically significant survival impact was found for the time between diagnosis and therapy initiation or for gender, surgery vs. primary C/RT, or pretherapeutic HNT presentation.

### 3.4. Subset Analysis of the T3/4 Cohort and Laryngectomy-Free Survival

Due to the uneven distribution of T3/4 patients between the HNT and non-HNT groups (125 vs. 18 patients), it is likely that a majority of T3/4 cases were already discussed by the interdisciplinary tumor board prior to certification and mandatory HNT presentation. In order to determine whether those patients, who were referred directly to therapy, possibly represented a more favorable subgroup, we conducted a subset analysis of the T3/4 cohort.

We found no statistically significant differences for age at initial diagnosis, gender, T-, N-, or R-status, recurrence, or therapy, including adjuvant therapy and neck dissections between the groups. Considering tumor localization, the HNT group presented significantly more glottic and supraglottic carcinomas (58 vs. 22%), while the non-HNT group showed a majority of laryngeal carcinoma with overlapping sites of the larynx (41 vs. 61%; *p* = 0.002). There was no M1 patient in the non-HNT group, whereas the HNT-group comprised 13 patients with distant metastasis (*p* < 0.001). All results are summarized in Table 7.

**Figure 8:**
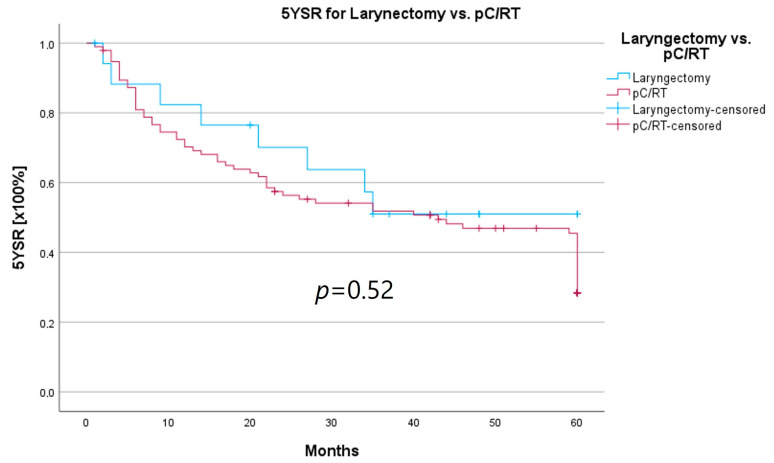
Comparison of 5YSR for laryngectomy vs. pC/RT.

**Figure 9:**
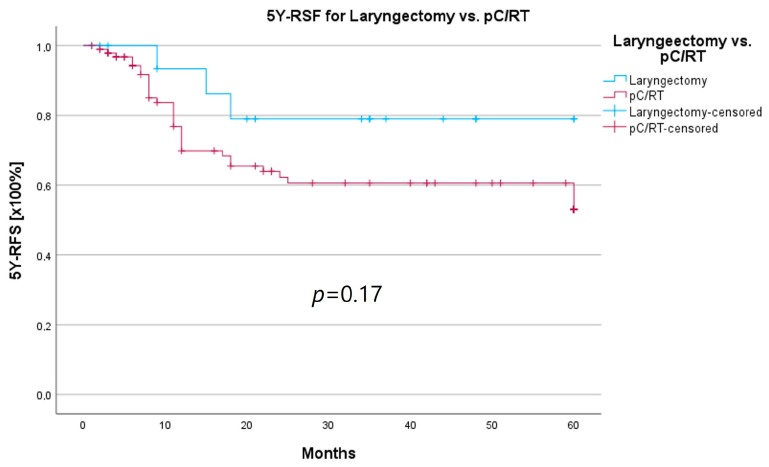
Comparison of 5Y-RFS for laryngectomy vs. pC/RT.

An important aspect of laryngeal carcinoma treatment is organ preservation while maintaining equal survival compared to non-organ-sparing treatment. The tumor board provides an opportunity to identify those patients who could benefit from a larynx-preserving form of therapy. As laryngectomy-free survival was an interesting parameter for treatment success and quality of life, but the non-HNT group consisted of only two patients who had received a laryngectomy, we assessed survival rates for both groups combined. Survival rates after laryngectomy were longer for both 5YSR and 5Y-RFS; however, they did not reach statistical significance (Table 8; Figure 8 and Figure 9).

## 4. Discussion

For the therapeutic management of laryngeal carcinoma, various surgical, radiotherapeutic, and haemato-oncological treatment modalities are available. The type and combination of therapy are dependent on the given tumor stage. At the same time, the functional outcome as well as patient’s wishes must be taken into account. Pretherapeutic head and neck tumor boards have been introduced to discuss the optimal treatment regimen and establish a guideline-based cancer therapy. In the presence of various specialist disciplines, the optimal treatment plan is developed, and diagnostic and staging decisions are made. Through tumor board presentation, not only interdisciplinary cooperation should be facilitated, but also patient quality care and survival should be improved [6]. Advanced tumor stages, which often require multimodal therapy concepts, are to benefit from tumor board presentation in particular [4]. In addition, the holding of head and neck tumor boards and the presentation of at least 95% of all primary tumor cases are important criteria for clinics to be certified as head and neck cancer centers [5]. The certificate confirms treatment at the highest quality level and is intended to contribute to the improvement of diagnostics, therapy, and follow-up care for tumor patients [6]. Certification requires the fulfilment of certain quality indicators, which are derived from the recommendations of evidence-based guidelines. Therefore, certified centers help to implement guideline-based therapy and improve the quality of treatment. For breast cancer patients treated at a certified center, a more positive course of disease as well as improvement in survival and guideline adherence has been shown at various times [7,8]. Also, for HNSCC patients, a survival benefit could be determined when treated at a certified head and neck cancer center [9]. In summary, a question arises regarding which of the required quality factors determine the survival advantage of therapy at a certified center. Is it an individual factor, such as the tumor board alone, or is it a combination of several factors?

The Head and Neck Cancer Center of the University Medical Center Freiburg achieved certification in 01/2015. From this point on, it also became mandatory to discuss primary tumor cases in a pretherapeutic head and neck tumor board. Prior to 2015, the presentation of primary tumor cases within a head and neck tumor board was facultative.

Our retrospective data analysis of 412 laryngeal cancer patients treated between 01/2010 and 12/2020 showed 98 patients who received therapy initiation without prior tumor board presentation. At the same time, this group showed significantly fewer advanced tumor stages when compared to the HNT group. This seems conclusive, because only complex tumor cases were presented to an HNT when tumor board presentation was not yet obligatory. Considering differences in therapy, a significantly larger proportion of primary C/RT in the HNT group can be found. T1/2 laryngeal carcinoma can be treated by surgery or primary radiotherapy, and survival is reported to be equal [10]. Treatment strategies for T3/4 carcinoma are either surgical resection with adjuvant C/RT or primary C/RT with possible salvage-laryngectomy or induction chemotherapy followed by surgery or radiotherapy [2]. In conclusion, the higher percentage of primary C/RT in the HNT group can be attributed to the also larger amount of advanced tumor stages in this group. In order to gain a more precise view of the influence of a pretherapeutic HNT on early-stage laryngeal carcinoma, differences in therapy for only T1/2 carcinoma were analyzed in a further step. Here, the significantly higher percentage of primary C/RT was depicted in the HNT group (31% vs. 19%, *p* = 0.027). This indicates that without a pretherapeutic tumor board presentation, a clear preference for primary surgical therapy, especially in early tumor stages, exists. In conclusion, pretherapeutic tumor board presentation led to the discussion of therapy alternatives apart from primary surgical therapy and a broader counselling of patients regarding their therapy options. A study by Gabel et al. also found that patients presented in a multidisciplinary breast board felt significantly better informed and accepted the proposed treatment more readily [11].

Regarding survival, 5YSR was shown to be similar for tumors with and without a pretherapeutic tumor board, although the HNT group presented significantly more advanced tumor stages. Consequently, this would imply that performing a pretherapeutic HNT for advanced tumor stages confers a survival benefit. However, survival analysis of only advanced tumor stages with or without pretherapeutic HNT showed no significant difference (33.7 vs. 32.7 months, *p* = 0.62). As a matter of fact, the non-HNT group consisted of only 18 patients with advanced tumor stages when compared to the HNT group with 125 patients, suggesting that even before pretherapeutic tumor board presentation was mandatory, more complex cases were already discussed in an interdisciplinary HNT. This fact makes it difficult to prove a survival benefit in our study. Friedland et al. compared 726 primary head and neck cancer patients and were able to show a significant improvement in 5-year survival rates for stage-IV patients managed in a multidisciplinary team setting [4]. Considering 5Y-RFS, the non-HNT group showed slightly better recurrence-free intervals without achieving statistical significance. There were no statistically significant differences regarding the number of recurrences between the groups.

In order to examine whether the 18 T3/4 patients who were not presented in a pretherapeutic tumor board comprised a more favorable subgroup, we performed a subset analysis of the T3/4 cohort. Indeed, we found a significantly higher proportion of distant metastasized patients (10 vs. 0%; *p* < 0.001) in the HNT group, as well as two patients with a unresectable T4b stage. This underlines the important role that the tumor board plays through the possibility of an interdisciplinary case discussion when it comes to more complex, advanced tumor stages. Still, regarding the therapy regimen in advanced laryngeal carcinoma, there was no significant difference between the groups. Laryngectomy was similarly distributed between the groups, with 15 vs. 11%, as was pC/RT, with 66 vs. 72%. Another important task of the HNT, particularly in the treatment of advanced laryngeal carcinomas, is to identify those patients who can benefit from organ-preserving therapy with equal survival rates. According to the German S3 guideline, the patient accepts a higher recurrence rate with organ-preserving pC/RT, but has salvage surgery as a curative option and no overall survival disadvantage [2]. Our analysis of laryngectomy-free survival showed a longer 5YSR (39.7 vs. 36.1 months) and 5Y-RFS (50.4 vs. 40.9 months) after receiving laryngectomy, but it did not achieve statistical significance. At the same time, these results suggest that organ preservation can be achieved even without a significant decrease in survival. However, in order to be able to make a relevant statement in this regard, further aspects, such as pretherapeutic laryngeal functionality and functional outcome, as well as the need for salvage laryngectomy, would have to be assessed. A study by Dyckhoff et al. provides a detailed overview of what aspects laryngeal organ preservation studies should take into account [12]. As this was a retrospective data collection study, it was not possible to directly record the functional outcome and quality of life, which certainly is a limitation of this study.

Following the given treatment recommendation by the tumor board, it was interesting to assess adherence to board recommendations and actual treatment in a next step. A study by Alkasbi et al. evaluated tumor board adherence for any newly diagnosed HNSCC during 2018 at the University Hospital in Lille and found a deviation from recommended therapy in 8.4% of cases [13]. Hollunder et al. found a deviation of 9.3% from head and neck tumor board recommendations at the University Hospital Bonn [14]. Graessle et al. analyzed adherence to treatment in elderly HNSCC patients treated at the Charité university medicine Berlin and found 14% of cases that were non-adherent [15]. With only 18 cases (6%) not following the therapy recommended by the HNT in our study, a very good adherence was achieved overall.

The significant delay in therapy initiation due to the pretherapeutic tumor board presentation, on the other hand, appears immense (37.9 vs. 20.1 days, *p* = 0.008). Nevertheless, this did not seem to have a significant influence on survival, because it was not calculated as a survival-modifying factor in the Cox model, nor were there significant differences in survival between the HNT and non-HNT group. One of the main reasons for the delayed therapy initiation in the HNT group is certainly the equally significantly more frequent use of primary chemoradiotherapy. Before performing C/RT, dental sanitation is necessary on the one hand, as well as performing a planning CT scan and developing a radiation protocol on the other hand, which can result in treatment delay [16,17,18]. Also, other factors, such as public holidays, can delay tumor board presentation and thus the initiation of therapy. Another aspect of therapy delay is comorbidity, which is particularly frequent in head and neck cancer patients [19]. This also results in more incidental findings in the staging, which must be assessed further and can thus lead to a delay in therapy.

At the same time, it should be a priority to minimize the time between tumor diagnosis and therapy initiation. Delays in therapy can lead not only to a decrease in survival due to progressive tumor growth, but also to functional impairments, upstaging, and the necessity of therapy extension, which is especially relevant in the case of laryngectomy [17,20]. Awareness must be created within teams and clinics to avoid delays in therapy through tumor board presentation, and concepts must be developed to improve workflows. Aydinguel et al. addressed this problem and designed a mobile ad hoc tumor board scheduling [21]. The concept of one-stop neck lump clinics with on-site cytology was also shown to accelerate diagnosis and early management [22]. When considering the time frame between initial diagnosis (which, in our study, was defined as the day of panendoscopy) and tumor board presentation, it is also noticeable that the mean time span is 12.3 days despite the weekly holding of tumor boards. This observation would also suggest the establishment of an automated notification system in the presence of malignant histological findings or the implementation of on-site cytology.

Apart from treatment delay, there are a number of other factors that can influence survival outcomes in laryngeal carcinoma and other HNSCC. Factors favoring survival are certainly treatment at a certified center; from a surgical point of view, the experience of the surgeon; from a radiotherapeutic point of view, compliance with the radiation protocol and the avoidance of interruptions; regular tumor follow-up care; and abstinence from noxious substances, like nicotine or alcohol consumption [16,23,24]. Calculations of our Cox model also identified age at initial diagnosis and number of secondary malignancies as survival-modifying parameters apart from UICC stages.

Considering pretherapeutic tumor board presentation, no survival-modifying effect was calculated in our study. Nonetheless, the holding of tumor boards and multidisciplinary head and neck cancer care play an important role in establishing guideline-based cancer therapy, optimizing diagnostic work-up, and follow-up care [25]. Different perspectives on therapy options and functionality are brought together, and radiological findings are discussed again on an interdisciplinary basis. A study by Kelly et al. found that after receiving multidisciplinary team care, HNSCC patients showed higher rates of dental assessment, nutritional assessment, PET staging, and use of adjuvant C/RT [26]. Moreover, tumor boards serve as a teaching event for doctors in training. In addition, it can be deduced from our results that tumor board presentation led to better counselling of patients regarding their therapy options.

## 5. Conclusions

The holding of pretherapeutic tumor boards is an important criterion for clinics to be certified as head and neck tumor centers. Tumor board presentation in a multidisciplinary team setting is supposed to improve interdisciplinary collaboration and survival outcomes, especially in advanced tumor stages. Our analyses of 412 laryngeal carcinoma patients compared survival and other epidemiological parameters between patients who were discussed by a pretherapeutic tumor board and patients who received therapy initiation without prior tumor board presentation. We found a significantly larger amount of primary C/RT for early tumor stages in the HNT group. These findings suggest that the HNT leads to a broader recognition of possible treatment options and, consequently, more elaborate patient counselling. Furthermore, the HNT group showed a significantly larger proportion of advanced tumor stages, suggesting that even before pretherapeutic tumor board presentation was mandatory, the HNT was used to discuss complex cases in an interdisciplinary setting. For this reason, the non-HNT group consisted of only 18 T3/4 patients when compared to the HNT group with 125 T3/4 patients, which made it difficult to provide a significant comparison study considering survival of the advanced tumor stages. On the other hand, we found a significant delay in treatment initiation in the HNT group. For one, this can be attributed to the larger amount of primary C/RT in this group, but, at the same time, this should create awareness for minimizing the time between diagnosis, tumor board presentation, and therapy initiation within teams and clinics. 

Overall, the holding of head and neck tumor boards plays an important part in facilitating interdisciplinary cooperation, especially in complex cases, the implementation of guideline-based cancer therapy, and, at the same time, providing a learning effect for doctors in training. However, the results of this study point out that there is still room for improvement of workflows in order to avoid delays in therapy.

## Figures and Tables

**Table 1:** Epidemiological data.

	Pretherapeutic HNT	No Pretherapeutic HNT	*p*-Value
*N*	314	98	
Age at initial diagnosis (years)			
Mean ± SD (Median)	65.8 ± 11 (66)	64.2 ± 10.6 (64)	0.21
Gender, *n* (%)			
Male:Female	265 (84)/49 (16)	88 (90)/10 (10)	0.15
Tumor localization, *n* (%)			0.89
Glottis	174 (55)	64 (65)	
Supraglottis	74 (24)	13 (13)	
Subglottis	8 (3)	1 (1)	
Laryngeal cartilage	1 (0.3)	1 (1)	
Overlapping sites of larynx	56 (18)	15 (15)	
Larynx, unspecified	1 (0.3)	4 (4)	
T status, *n* (%)			<0.001
T1	143 (46)	58 (59)	
T2	45 (14)	22 (22)	
T3	57 (18)	6 (6)	
T4	68 (22)	12 (12)	
N status, *n* (%)			0.15
N0	211 (67)	70 (71)	
N1	13 (4)	5 (5)	
N2	81 (26)	20 (20)	
N3	8 (3)	1 (1)	
M status, *n* (%)			0.23
M0	295 (94)	94 (96)	
M1	18 (6)	3 (3)	
UICC classification, *n* (%)			0.003
I	138 (44)	55 (56)	
II	25 (8)	16 (15)	
III	34 (11)	5 (5)	
IVA	59 (19)	11 (11)	
IVB	39 (12)	9 (9)	
IVC	18 (6)	3 (3)	
R status, *n* (%)			0.4
R0	126 (87)	53 (83)	
R1	19 (13)	11 (17)	
Recurrence, *n* (%)			0.41
None	244 (78)	80 (82)	
Local	41 (13)	12 (12)	
Regional	8 (3)	-	
Distant	4 (1)	3 (3)	
Locoregional	12 (4)	2 (2)	
Loco distant	3 (1)	-	
Regional and distant	1 (0.3)	1 (1)	
Locoregional and distant	1 (0.3)	-	
Secondary tumors, *n* (%)			0.56
0	257 (82)	71 (72)	
1	51 (16)	22 (22)	
2	4 (1)	4 (4)	
3	2 (0.6)	-	
5	-	1 (1)	

**Table 2:** Treatment regimen.

	Pretherapeutic HNT	No Pretherapeutic HNT	*p*-Value
Therapy, *n* (%) Surgery pC/RT			<0.001
	145 (46)	64 (65)	
	140 (45)	28 (29)	
Best supportive care	18 (6)	3 (3)	
Adjuvant therapy, *n* (%)	25 (17)	10 (16)	0.77
Delta diagnosis > HNT presentation (days)			
Mean ± SD (Median)	12.3 ± 22.9 (10)	-	
Delta diagnosis > therapy initiation (days)			0.008
Mean ± SD (Median)	37.9 ± 62 (27)	20.1 ± 19 (15)	
Delta diagnosis > surgery (days) Mean ± SD (Median)	20.6 ± 12.6 (20)	11.8 ± 9 (12)	<0.001
Delta diagnosis > pC/RT (days) Mean ± SD (Median)	54.7 ± 81.6 (41)	40.7 ± 22 (38)	0.4
Non-tumor-board-adherent cases, *n* (%)	18 (6)	-	
Patient’s wish	11 (4)	-	
Intra-clinic decision	4 (1)	-	
Unknown	3 (1)	-	

**Table 3:** Differences in therapy regimen for T1/2 stages.

	Pretherapeutic HNT	No Pretherapeutic HNT	*p*-Value
Therapy, *n* (%)			0.027
Surgery	122 (65)	61 (76)	
pC/RT	59 (31)	15 (19)	

**Table 4:** Survival data.

	Pretherapeutic HNT	No Pretherapeutic HNT	*p*-Value
5-Year Survival Rate (5YSR) (months)			0.96
Mean ± SD	43.4 ± 1.3	47 ± 2	
5-Year Recurrence-Free Survival (5Y-RFS) (months)			0.16
Mean ± SD	48.2 ± 1.3	51.9 ± 1.8	

**Table 5:** Survival data grouped by T status.

	Pretherapeutic HNT	No Pretherapeutic HNT	*p*-Value
5YSR for T1/2 (months)			0.19
Mean ± SD	49.7 ± 1.4	50.2 ± 2	
5YSR for T3/4 (months)			0.62
Mean ± SD	33.7 ± 2.3	32.7 ± 5.7	
5Y-RFS for T1/2 (months)			0.55
Mean ± SD	50.8 ± 1.4	52.9 ± 1.9	
5Y-RFS for T3/4 (months)			0.49
Mean ± SD	42.6 ± 2.5	44.7 ± 6.2	

**Table 6:** Forward Cox regression.

Variable	Hazard Ratio	95% Confidence Interval	*p*-Value
Age at initial diagnosis	1.051	1.034–1.069	<0.001
UICC classification UICC I–II vs. UICC III–IV	2.954	2.125–4.107	<0.001
Secondary tumors	1.241	1.009–1.525	0.04

**Table 7:** Subset analysis of the T3/4 cohort.

	Pretherapeutic HNT	No Pretherapeutic HNT	*p*-Value
*N*	125	18	
Age at initial diagnosis (years)			0.82
Mean ± SD	65.7 ± 10.9	66.4 ± 7.6	
Gender, *n* (%)			0.6
Male:Female	109 (87)/16 (13)	15 (83)/3 (17)	
Tumor localization, *n* (%)			0.002
Glottis	32 (26)	2 (11)	
Supraglottis	40 (32)	2 (11)	
Subglottis	2 (2)	-	
Laryngeal cartilage	-	-	
Overlapping sites of larynx	51 (41)	11 (61)	
Larynx, unspecified	-	3 (17)	
T status, *n* (%)			0.48
T3	55 (44)	6 (33)	
T4a	68 (54)	12 (67)	
T4b	2 (2)	-	
N status, *n* (%)			0.64
N0	48 (38)	4 (22)	
N1	6 (5)	3 (17)	
N2	66 (53)	11 (61)	
N3	5 (4)	-	
M status, *n* (%)			<0.001
M0	112 (90)	18 (100)	
M1	13 (10)	-	
R status, *n* (%)			0.22
R0	20 (87)	2 (67)	
R1	3 (13)	1 (33)	
Recurrence, *n* (%)			0.75
None	72 (67)	7 (64)	
Local	17 16)	1 (9)	
Regional	2 (2)	-	
Distant	3 (3)	2 (18)	
Locoregional	8 (8)	1 (9)	
Loco distant	3 (3)	-	
Regional and distant	1 (1)	-	
Locoregional and distant	1 (1)	-	
Therapy, *n* (%)			0.69
Laryngectomy	19 (15)	2 (11)	
Partial laryngectomy	4 (3)	1 (6)	
pC/RT	82 (66)	13 (72)	
Best supportive care	14 (11)	1 (6)	
Unknown/no therapy	6 (5)	1 (6)	
Adjuvant therapy, *n* (%)	17 (74)	3 (100)	0.97
Neck Dissection, *n* (%)	21 (91)	3 (100)	0.76

**Table 8:** Laryngectomy-free survival.

	Laryngectomy	pC/RT	*p*-Value
*N*	21	95	
5YSR (months)			0.52
Mean ± SD	39.7 ± 5.5	36.1 ± 2.5	
5Y-RFS (months)			0.17
Mean ± SD	50.4 ± 5	40.9 ± 2.8	

## Data Availability

The data presented in this study are available in this article.
